# Study of an ${\hbox {MoS}}_{2}$ phototransistor using a compact numerical method enabling detailed analysis of 2D material phototransistors

**DOI:** 10.1038/s41598-024-66171-1

**Published:** 2024-07-03

**Authors:** Raonaqul Islam, Ishraq Md. Anjum, Curtis R. Menyuk, Ergun Simsek

**Affiliations:** Department of Computer Science and Electrical Engineering, University of Maryland Baltimore County, Baltimore, MD 21250 USA

**Keywords:** Electrical and electronic engineering, Other photonics

## Abstract

Research on two-dimensional material-based phototransistors has recently become a topic of great interest. However, the high number of design features, which impact the performance of these devices, and the multi-physical nature of the device operation make the accurate analysis of these devices a challenge. Here, we present a simple yet effective numerical framework to overcome this challenge. The one-dimensional framework is constructed on the drift-diffusion equations, Poisson’s equation, and wave propagation in multi-layered medium formalism. We apply this framework to study phototransistors made from monolayer molybdenum disulfide (${\hbox {MoS}}_{2}$) placed on top of a back-gated silicon-oxide-coated silicon substrate. Numerical results, which show good agreement with the experimental results found in the literature, emphasize the necessity of including the inhomogeneous background for accurately calculating device metrics such as quantum efficiency and bandwidth. For the first time in literature, we calculate the phase noise of these phototransistors, which is a crucial performance metric for many applications where precise timing and synchronization are critical. We determine that applying a low drain-to-source voltage is the key requirement for low phase noise.

## Introduction

Over the past two decades, we have witnessed a growing interest in using two-dimensional (2D) materials for a variety of photonic and optoelectronic applications^1–17^ due to their promising optical, electrical, thermal, and mechanical properties^1,3,6–10,18–20^. In parallel, material scientists have developed practical methods such as chemical vapor deposition synthesis^18^, liquid exfoliation^21^, and laser thinning^22^ to grow these materials with desired properties^23^.

Monolayers of transition metal dichalcogenides have been a widespread choice for detecting light due to their direct bandgap and moderate absorption in the visible part of the electromagnetic spectrum^1,3,6,7^. Molybdenum disulfide (${\hbox {MoS}}_{2}$) based phototransistors is a mature research subject that has been discussed in several publications^2,8,11–16,24–35^. Yin et al. introduced a phototransistor based on a single-layer ${\hbox {MoS}}_{2}$ with a photoresponsivity of 7.5 mA/W under low incident power and moderate gate voltage conditions, surpassing the performance of graphene-based counterparts^2^. Lopez-Sanchez et al. exhibited an extension of photoresponsivity to an impressive 880 A/W with a 561 nm laser incident^24^. Other investigations delved into photoconductive and photogating effects^8,11^, as well as intrinsic optoelectronic traits^19^. Meanwhile, Lan et al. demonstrated the attainment of ultra-high photoresponsivity (approximately $2.7 \times 10^4$ A/W) by integrating two-dimensional plasmonic crystals with the ${\hbox {MoS}}_{2}$ field-effect transistor^12^. A buried gate device has been proposed to eliminate the reliance on high gate voltage tuning^13^. Moreover, Luo et al. disclosed the achievement of near-infrared photoresponse through a heterojunction formed by ${\hbox {MoS}}_{2}$ and 2D-polyimide^14^. Further explorations encompass a waveguide-integrated photodetector operating at the telecom band^17^, avalanche phototransistors^15^, and pixel sensor matrices^16^. Some other researchers investigated the influence of the ambient temperature on the device performance and reported that the photo-current increases with temperature despite decreasing mobilities^26,34,36^. They attributed this phenomenon to either defect traps^26^ or oxygen desorption^34,36^.

The ability of a ${\hbox {MoS}}_{2}$-based phototransistors to convert optical excitations to electrical currents depends on several factors such as the quality, dimensions, doping and defect level of the 2D material, the materials used in the substrate, and their thicknesses, the type of the metal used for contacts, the shape and location of the contacts, ambient temperature, wavelength and strength of the optical excitation, and applied voltages. Due to having such a high number of variables, using numerical methods can be more accurate than using approximate analytical formulas to design application-specific phototransistors. In this direction, Ueda et al. analyzed the carrier distributions along a monolayer tungsten diselenide (WSe_2_) transistor covered with an ionic liquid by solving the drift-diffusion equations in two-dimensions^37^. To our knowledge, their work is the first that focuses on ion-gated transistor devices made from 2D materials. In another study, Chen et al. introduced a numerical approach that combined the drift-diffusion transport equations with a two-dimensional (2D) Poisson equation in order to simulate a 2D device structure^38^. Both studies have presented results that agree with experimental results found in the literature. With this study, we would like to extend their efforts and present a simple yet accurate and complete formulation that allows advanced characterization of 2D material-based phototransistors. The method relies on the drift-diffusion equations combined with Poisson’s equation and wave propagation in a multi-layered media formalism. The material properties are determined realistically with the help of numerical material models based on experimental data. In addition to calculating the output current as a function of incident power, wavelength, device dimensions, and temperature, we also calculate other critical performance metrics, such as bandwidth and phase noise. Our numerical results confirm previously reported experimental results, such as the highest quantum efficiency is obtained at the wavelength of 561 nm, whereas the strongest photocurrent is observed at lower wavelengths ($\sim$ 425 nm). On the other hand, our numerical analysis also reveals some new observations. For example, the lowest phase noise is achieved at 475 nm, somewhere in between where we have the highest quantum efficiency and largest photocurrent, indicating the trade-off between quantum efficiency and response time. Our study also helps us understand why quantum efficiency drops with temperature while photocurrent increases. Similar to these, we obtained many other results numerically. Let us discuss them first. Then, we provide all the main details of our methods and models.

## Results and discussion

**Figure 1:**
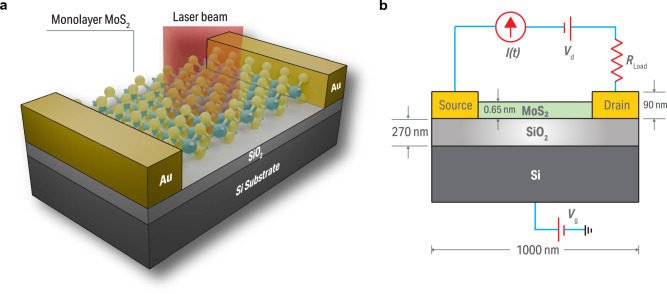
Schematic diagram of the phototransistor structure in (**a**) a three-dimensional view with an incident light beam, (**b**) a two-dimensional view with dimensions and circuitry.

Figure 1 displays the device configuration that we study. A 0.65 nm thick monolayer of ${\hbox {MoS}}_{2}$ is positioned atop a 270 nm thick silicon dioxide (SiO$_2$) layer. Under the SiO$_2$, we have a 2 $\upmu$m thick, back-gated silicon (Si) substrate. The gold contacts for the source and drain are positioned on two sides of the 2D material. The ${\hbox {MoS}}_{2}$ is assumed to be 1 $\upmu$m long. Both the gates and ${\hbox {MoS}}_{2}$ layer are assumed to be 1 $\upmu$m wide to determine the carrier concentrations per unit area and total number of photons that enter the device. The device is illuminated from above. The device operates as a field-effect transistor in which the ${\hbox {MoS}}_{2}$ monolayer functions as a semiconducting channel.

For all the results presented here, we use a uniform 1D mesh, dividing the ${\hbox {MoS}}_{2}$ layer into 1000 1D elements with a width of 1 nm. The temperature is assumed to be constant, $T = 300$ K, in all our results except in our final study, in which we investigate the effect of temperature on the quantum efficiency and phase noise. The drain contact is linked to the bias voltage, while the source contact is connected to the ground.

**Figure 2:**
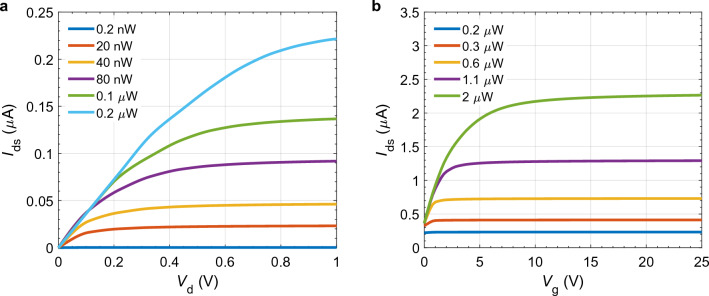
(**a**) Output current in ${\hbox {MoS}}_{2}$ layer at different drain voltages (${V}_\text{d}$) when gate voltage $V_\text{g}=0\;V$ and (**b**) different ${V}_\text{g}$ when $V_\text{d}=1\;V$ with varying incident power $P_{\text{in}}$ at $\lambda = 561\;\text {nm}$ and 300 K temperature.

We begin with a steady-state analysis in which we assume that the phototransistor is illuminated with continuous laser light with a wavelength equal to 561 nm. The incident power of the laser is controlled by varying the $P_0$ factor, described in the “Methods” section. First, we set the gate voltage $V_\text{g}$ to zero. We then increase the source-to-drain voltage ($V_{d}$) linearly from 0 V to 1 V, and we calculate the output current, $I_{ds}$, assuming six different incident power levels ranging from 0.2 nW to 0.2 $\upmu$W. As shown in Fig. 2a, the intensity of incident light and the source-to-drain voltage influence the output current. When a small bias voltage is applied in the presence of no or extremely weak illumination, the free electrons generated due to doping tend to migrate toward the drain contact. At the same time, the holes predominantly accumulate near the source contact of the ${\hbox {MoS}}_{2}$ monolayer. As we increase $V_\text{d}$, the electric field across the ${\hbox {MoS}}_{2}$ film increases. This field increase enhances the collection of charge carriers generated by the incident light, and as a result, the output current first increases with this increase in $V_\text{d}$ and then saturates. Increasing the strength of the optical excitation increases the number of electron–hole pairs that are generated, which leads to an increase in the output current since more charge carriers are available for transport across the device.

For our following steady-state characterization, we set the source-to-drain voltage to 1 V and incrementally increase the gate voltage from 0 to 25 V. We compute the output current at five incident powers ranging from 0.2 to 2 $\upmu$W. As shown in Fig. 2b, the output current is influenced by both the intensity of incident light and the gate voltage. As expected, increasing the gate voltage affects the number of carriers and, hence, the conductivity of the channel between the source and drain terminals up to a certain point. Similar to the first study, increasing the strength of the optical excitation increases the number of electron–hole pairs that are generated, which leads to an increase in the output current. The results of these steady-state studies agree with the experimentally measured results found in the literature^19,24,39^.

Next, we present two sets of time domain calculations. For both sets, the time stepping of the dynamic solver, $\Delta t$, is chosen as 10 ps, and the total execution time is fixed to $200 \ \upmu$s. The reasons why we have chosen these values are discussed in the “Methods” section in more detail.

**Figure 3:**
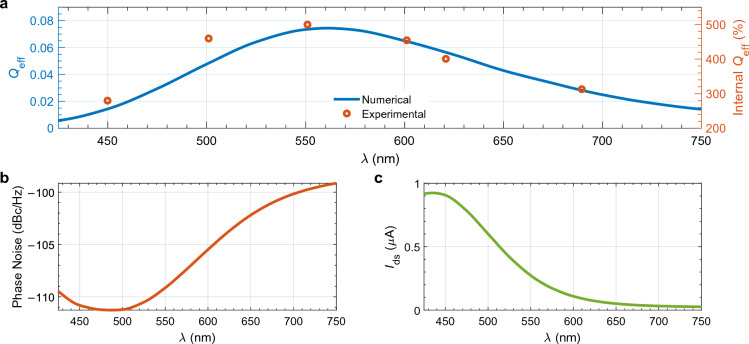
(**a**) Comparison of the total quantum efficiency (left y-axis) and internal quantum efficiency^19^ (right y-axis), (**b**) calculated phase noise of the device, and (**c**) its output current as functions of wavelength at $V_\text{g}=10\;V$, $V_\text{d}=0.5\;V$, and $P=0.2 \ \upmu$W.

To obtain the quantum efficiency (${Q}_{\text{eff}}$) of the phototransistor as a function of wavelength, we calculate the total number of photons ($N_\mathrm{{in}}$) that enter the device and the total number of electrons ($N_\mathrm{{out}}^\mathrm{{ill}}$) generated during the entire illumination at 14 wavelengths ranging from 425 to 750 nm. We calculate the total number of of electrons ($N_{out}^{dark}$) when there is no illumination. Hence, $Q_\mathrm{{eff}} = (N_\mathrm{{out}}^\mathrm{{ill}}-N_{out}^{dark})/N_\mathrm{{in}}$. We set $V_\text{g}$ and $V_\text{d}$ to 10 V and 0.5 V, respectively, and we set the incidence power to 0.2 $\upmu$W. The result presented in Fig. 3a shows that the ${Q}_{\text{eff}}$ highly depends on the electric field intensity experienced by the ${\hbox {MoS}}_{2}$ film. As explained in the Supplementary Materials, the electric field intensity also peaks around 561 nm, making MoS$_2$-based phototransistors particularly suitable for applications in this part of the visible spectrum. In Fig. 3a, we also show the experimentally measured internal quantum efficiency (*IQ*_eff_)^19^ on the right *y*-axis. The difference between *IQ*_eff_ and ${Q}_{\text{eff}}$ is that *IQ*_eff_ calculation only considers the absorbed photons, while ${Q}_{\text{eff}}$ calculation considers all the incident photons. That is why the scales are different. Moreover, the structure from^19^ has a heterostructure that includes a hexagonal boron nitride layer in between the monolayer ${\hbox {MoS}}_{2}$ and the SiO$_2$ layer, which enhances the absorption of photons in the ${\hbox {MoS}}_{2}$ layer. This mismatch between the structure in^19^ with our studied device creates some discrepancy in the scaling factor. Nevertheless, we show that our numerically calculated ${Q}_{\text{eff}}$ accurately matches the experimentally measured ${Q}_{\text{eff}}$ with proper scaling. We should also note that for a thick Si substrate, the main factor determining the electric field intensity inside the monolayer ${\hbox {MoS}}_{2}$ as a function of wavelength is the thickness of the SiO$_2$ layer. When this value equals odd multiples of 90 nm (i.e., 90 nm, 270 nm, or 450 nm), it yields the highest intensity^40^. As a result, these thicknesses lead to the highest possible contrast between the ${\hbox {MoS}}_{2}$ coated and bare substrates, which makes ${\hbox {MoS}}_{2}$ more visible and makes it easier for researchers to locate the ${\hbox {MoS}}_{2}$ covered region on the substrate. In an experimental study by Mukherjee et al.^41^, the thickness of the SiO$_2$ layer was 300 nm, and they observed that the quantum efficiency peaks at 540 nm, a slightly shorter wavelength. In short, if one wants to implement a drift-diffusion model for characterizing 2D material-based phototransistors, then the inhomogeneous background needs to be taken into account. One easy yet efficient way of achieving this is following the wave propagation in layered media formalism.

**Figure 4:**
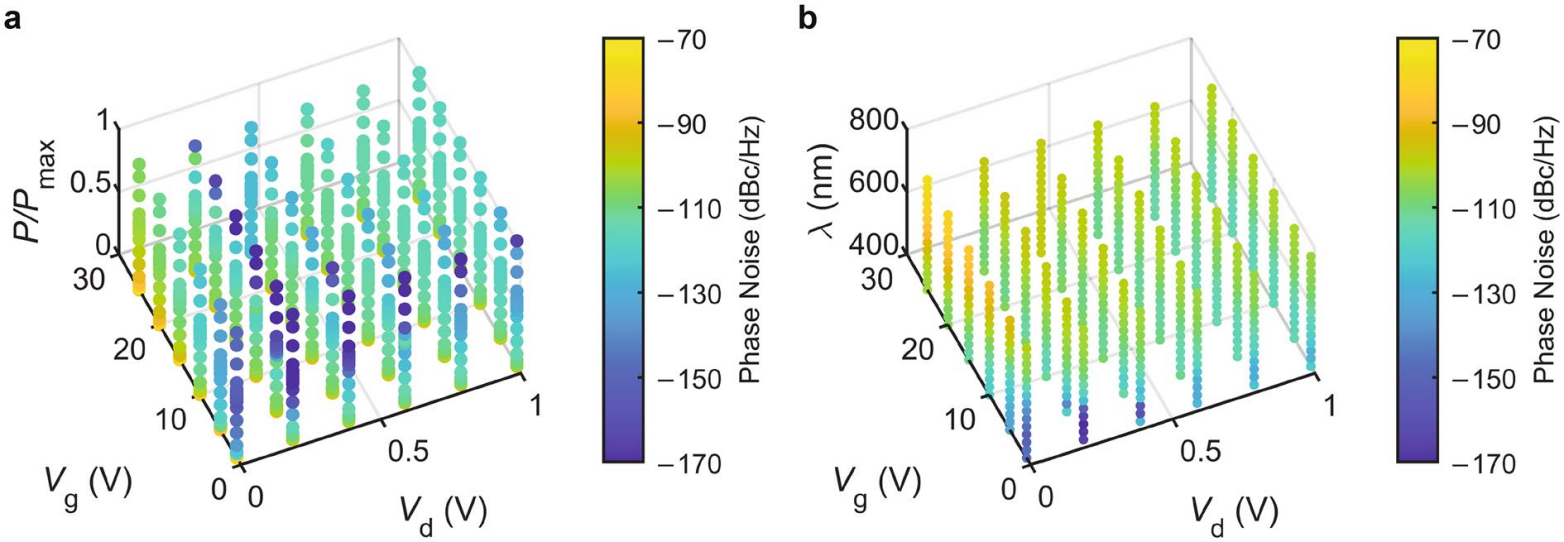
Phase noise as functions of gate voltage ($V_g$), drain-to-source voltage ($V_d$), and (**a**) normalized incident power $P/P_\mathrm{{max}}$ and (**b**) wavelength ($\lambda$).

One might naively expect to have the largest output current when the excitation wavelength is equal to 561 nm, and since the phase noise generally decreases with increasing current, an expectation of having the least phase noise at the same wavelength would not be inconsistent. In Fig. 3b,c, we plot the calculated values of phase noise and output current as a function of incidence wavelength. As expected, they exhibit opposite behaviors: the larger the current, the lower the phase noise. However, the wavelength where we observe the highest output current (or the lowest phase noise) is not the same as the one where we observe the highest quantum efficiency due to stronger absorption of ${\hbox {MoS}}_{2}$ at these lower wavelengths^6,42^. To our knowledge, our work is the first to calculate the phase noise of a 2D-material-based phototransistor. For $V_\text{g}=10\;V$, $V_\text{d}=0.5\;V$, and $P=0.2 \ \upmu$W. the phase noise varies between $- 112$ dBc/Hz and $- 99$ dBc/Hz, which is 70 dBc/Hz higher than state-of-the-art photodetectors^43,44^. To examine how the phase noise changes with incident power, wavelength, and voltages, we carry out additional sets of calculations. The results illustrated in Fig. 4 shows that the idea drain-to-source voltage value is close to 0.2 V. For this $V_d$ value, it is possible to achieve a phase noise value as low as $- 170$ dBc/Hz when $\lambda$ is close to 475 nm, $V_g$ is less than 20 V, and incident power is at $\mu$W level.

One might try to further lower the phase noise by placing metal nanoparticles near the 2D material. If their dimensions and inter-particle spacing are carefully chosen, these particles (made from metals such as gold and silver that have a negative permittivity and low loss in the visible part of the electromagnetic spectrum) can enhance the local electric field, increase the output current, and help reduce the phase noise. Another strategy could be making changes in the substrate design to remove the heat from the 2D material more effectively. As discussed later, the phase noise decreases with decreasing temperature.

**Figure 5:**
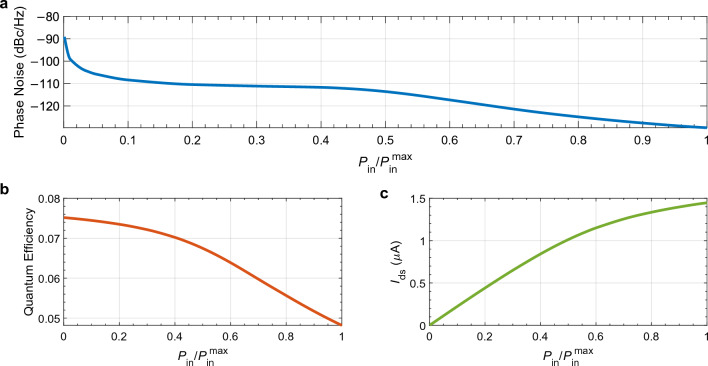
(**a**) Phase noise, (**b**) overall quantum efficiency, and (**c**) output current of the ${\hbox {MoS}}_{2}$ phototransistor calculated as functions of incident power ratio where *P* varies from 2 nW to 2 $\upmu$W at $V_\text{g}=10\;V$, $V_\text{d}=0.5\;V$, and $\lambda =561\;\text{nm}$.

Next, we study how the incident power affects the phase noise, ${Q}_{\text{eff}}$, and output current. We set the excitation wavelength to 561 nm to match the point of highest ${Q}_{\text{eff}}$, as shown in Fig. 3a. The gate and source-to-drain voltages are set to 10 V and 0.5 V, respectively. Figure 5 shows the results. We observe that the phase noise and ${Q}_{\text{eff}}$ decay with increasing power while the output current increases. As expected, more photon energy is absorbed by the semiconductor material, leading to the generation of a more significant number of electron–hole pairs and, eventually, a higher output current. As the power increases, the semiconductor material eventually becomes saturated with carriers, so further increases in light power do not lead to a proportional increase in current, as observed in Fig. 5c. Since the phase noise generally decreases with increasing current, the result we present in Fig. 5a is unsurprising. Similar to the experimental results^12,24,39^, we also observe in Fig. 5b that the ${Q}_{\text{eff}}$ of the device decreases with incident power. Even though we provide more photons by increasing the excitation intensity, the increase in the generation of additional electron-hole pairs is not proportional due to the saturating output current, and hence, the ${Q}_{\text{eff}}$ decreases.

The results depicted in Fig. 3 show us that there is a clear trade-off between ${Q}_{\text{eff}}$ and phase noise. As the incident power increases, ${Q}_{\text{eff}}$ decreases while phase noise improves. Similarly, there is a trade-off between ${Q}_{\text{eff}}$ and output current. Higher incident power leads to higher output currents but lower ${Q}_{\text{eff}}$. Operating the phototransistor at higher incident powers is beneficial for applications requiring low phase noise and high output current. Conversely, operating at lower incident powers is preferable for applications where high ${Q}_{\text{eff}}$ (or responsivity) is critical.

**Figure 6:**
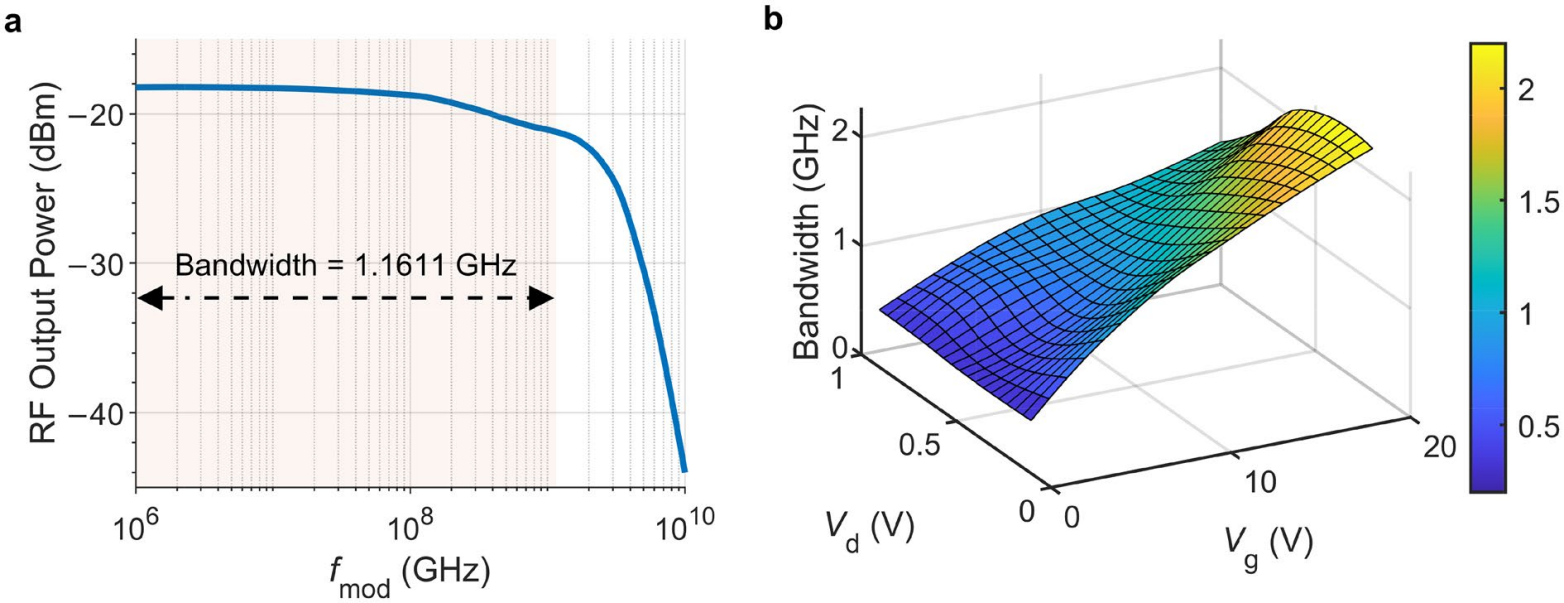
(**a**) RF output power of a $1 \ \upmu \text{m} \times 1 \ \upmu \text{m}$ ${\hbox {MoS}}_{2}$ phototransistor with a 5% modulation depth and modulation frequency ranged between 1 MHz and 10 GHz with $V_\text{g}=10\;V$, $V_\text{d}=0.5\;V$, $\lambda =561\;\mathrm nm$, and $T = 300$ K. (**b**) 3 dB bandwidth of the same photodetector as a function of $V_g$ and $V_d$.

Next, we calculate the phototransistor’s radio-frequency (RF) output power. For this section, we set the excitation wavelength to 561 nm and the gate and source-to-drain voltages to 10 V and 0.5 V, respectively. The incident power is 0.2 $\upmu$W. We modulate the excitation using the expression $G_\text{in} = G[1+m \sin {(2\pi f_{\text {mod}}t)}]$. We set the modulation depth equal to 50% ($m =0.5$), and we vary the modulation frequency ($f_\text {mod}$) logarithmically from 1 MHz to 10 GHz. We present the results in Fig. 6a, highlighting the 3 dB bandwidth with light shading. The computed bandwidth of 1.16 GHz is lower than 1.37 GHz, reported by Zhiwen et al.^17^ for a similar device fabricated on a slightly different substrate (Si$_3$N$_4$/SiO$_2$/Si). Figure 6b shows the 3 dB bandwidth of the same photodetector as a function of gate and drain voltages. The highest bandwidth is obtained with high $V_g$ and moderate $V_d$. The dependency of the bandwidth on $V_g$ is straightforward. When we increase $V_g$, the electric field at the gate induces more charge carriers in the monolayer MoS$_2$ channel, and this increased carrier density improves the conductivity of the channel. More carriers mean the transistor can support higher current flow, reducing the *RC* time constants associated with the transistor, where *R* is the resistance and *C* is the capacitance. Lower *RC* time constants result in higher bandwidth. The influence of $V_d$ is more complicated. At lower values of $V_d$, increasing the drain voltage increases the electric field along the channel, accelerating the electrons. This acceleration improves carrier mobility and reduces channel resistance, increasing bandwidth. After reaching the saturation point, the further increase in the drain voltage causes a decrease in the drift velocity and, hence, in the bandwidth.

We should also note that the bandwidth of the phototransistor also changes with dimensions. For example, the $10 \ \upmu \text{m} \times 10 \ \upmu \text{m}$ and $40 \ \upmu \text{m} \times 40 \ \upmu \text{m}$ photodetectors have the bandwidths of 195 MHz and 28.4 MHz, respectively. The larger phototransistors capture more light and exhibit large responsivities, but larger active areas also increase the capacitance. Higher capacitance slows down the *RC* time constant limiting the photodetector’s bandwidth.^45^

Table 1 provides a list of measured responsivity values of monolayer MoS$_2$-based photodetectors in chronological order. The reported responsivity values cover a giant range, from a $7.5 \times 10^{-3}$ A/W^2^ to $8.84 \times 10^{8}$ A/W^35^. One thing we observe immediately is the responsivity increases with decreasing optical power. This inverse dependency can be attributed to several mechanisms, such as photoconductive gain and trap states. For the former, fewer electron–hole pairs are generated when optical powers are low, and the recombination rate of these carriers is reduced. This results in longer carrier lifetimes, allowing more charge carriers to contribute to the photocurrent, effectively increasing the responsivity. For the latter, we know that monolayer MoS$_2$ has a high density of trap states (defects) in its band structure. At low light intensities, the generated carriers can get trapped in these states, which prolongs their lifetime because they are not immediately recombining. When the optical power is low, the traps are less likely to be saturated to capture and hold carriers longer. These trapped carriers can be subsequently released, contributing to a larger photocurrent over an extended period and thus increasing the overall responsivity at the expense of slow response time. Unfortunately, for most of the references mentioned in Table 1, we do not know the dimensions of the phototransistor. The responsivity value (3.64 $\times 10^{-2}$ A/W) that we calculate for a $1 \ \upmu \text{m}\times 1\ \upmu \text{m}$ phototransistor is close to the one ($7.5 \times 10^{-3}$ A/W) measured on a $2.1\ \upmu \text{m} \times 2.6\ \upmu \text{m}$ phototransistor^2^.

**Table 1:** Comparison of the monolayer MoS$_2$ based photodetectors’ responsivity in chronological order.

Reference	$\lambda$ (nm)	$V_d$ (V)	$V_g$ (V)	$P_{\text {inc}}$	Responsivity (A/W)
Yin et al.^2^	488	1	50	80 $\upmu$W	$7.5\times 10^{-3}$
Lopez-Sanchez et al.^24^	561	8	$-70$	1 $\upmu$W	20
Yang et al.^25^	635	1	20	0.1 nW	110
Kufer and Konstantatos^27^	550–675	5	$- 32$	32 mW	210–310
Dhyani and Das^28^	580	N/A	3	N/A	6.8
Sun et al.^29^	590	3	10	0.3 nW	60
Islam et al.^30^	450	1	3	35.18 mW	12.03
Li et al.^31^	460	N/A	20	1.4 $\upmu$W	16.1
Yang et al.^32^	633	N/A	60	5 $\upmu$W	3.4
Sahu et al.^33^	514.5	1	0	0.127 nW	47
Bartolomeo et al.^34^	571	0.5	3	17.5 pW	30
Schranghamer et al.^35^	450	5	$- 5$	0.15 pW	8.84 $\times 10^{8}$
This work: $1\ \upmu \text{m}\times 1\ \upmu \text{m}$ $40\ \upmu \text{m}\times 40\ \upmu \text{m}$	561	0.5	10	0.2 $\upmu$W	3.64$\times 10^{-2}$ 0.145

Some values provided in this table are approximate (extracted from the published figures).

**Figure 7:**
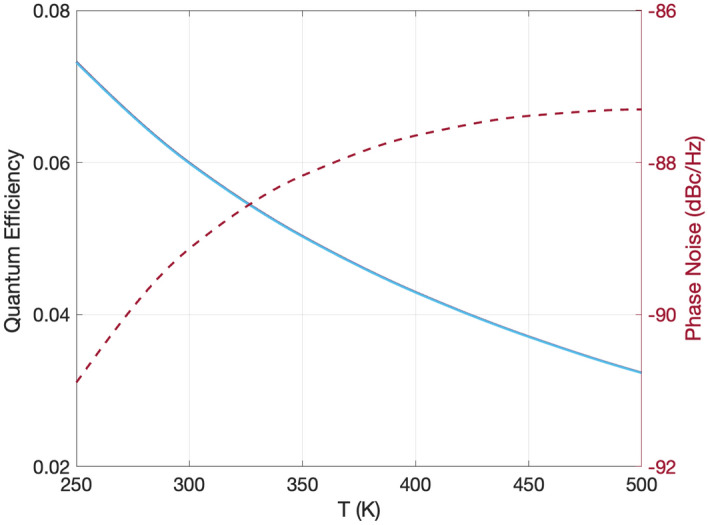
Quantum efficiency and phase noise as a function of local temperature (*T*), where $V_\text{g}=10\;V$, $V_\text{d}=0.5\;V$, $\lambda =561\;\mathrm nm$, and $P_{in} = 2$ nW.

Finally, we calculate the phototransistor’s ${Q}_{\text{eff}}$ and phase noise as a function of ambient temperature. Again, the wavelength of the excitation is 561 nm. The gate and source-to-drain voltages are 10 V and 0.5 V, respectively. The incident power is 2 nW. The ambient temperature is increased from 250 to 500 K uniformly. We show the results in Fig. 7. Even though the phase noise increases slightly from $- 90.9$ dBc/Hz to $- 87.3$ dBc/Hz, ${Q}_{\text{eff}}$ drops significantly from 7.32 to 3.23%. This result might look like a contradiction with respect to the experimental results^26,34^, where an increase in the photocurrent is observed with increasing temperature. Indeed, we observe a similar increase in the photocurrent. However, the increase in the dark current is more significant than the increase in the photocurrent; hence, the overall ${Q}_{\text{eff}}$ of the device drops with temperature.

It is numerically confirmed that the highest quantum efficiency is achieved at a wavelength of 561 nm under moderate optical excitation at room temperature when the oxide thickness is 270 nm. However, the wavelengths leading to the highest output current and lowest phase noise are different. Similar to those observed in experiments, we numerically show that the quantum efficiency decreases with increasing optical power and temperature. At high drain-to-source and gate voltages, the phase noise of the ${\hbox {MoS}}_{2}$-based phototransistors can be 70–80 dBc/Hz higher than that of state-of-the-art photodetectors, which limits their usability in applications requiring low phase noise and high output current. For a monolayer MoS$_2$ phototransistor fabricated over 270 nm thick SiO$_2$-coated with Si layer, we determine that the ideal $V_d$ value is 0.2 V, the $V_g$ should be less than 20 V, the wavelength should be close to 475 nm. Under these conditions, the phase noise can be as low as $- 170$ dBc/Hz, close to state-of-the-art photodetectors’ phase noise level^46^.

In conclusion, we have presented a compact numerical solution for 2D material phototransistors that takes a convenient 1D pathway. We have combined the drift-diffusion equations, realistic material modeling, and wave propagation in layered media formalism to conduct an efficient numerical study on the ${\hbox {MoS}}_{2}$ device. Our solver involves a practical approach to finding the vital effects of voltages, incident power, substrate, and the ambient temperature. We have numerically demonstrated the critical performance metrics of this class of devices, such as output current, quantum efficiency, phase noise, and RF output power, as functions of laser wavelength and illumination intensity. Our results are in good agreement with published experimental data.

## Methods

Here, we first provide a mathematical model that is valid for any type of photodetector/phototransistor, such as *p-i-n* photodetector, uni-wave traveling carrier photodetector, and avalanche photodiodes. Then, we explain the steps required for accurate simulation of phototransistors made from 2D materials and provide the details of our material modeling.

### Phototransistor/photodetector modeling

To simulate the carrier transport behavior of phototransistors and photodetectors, we employ a one-dimensional drift-diffusion model^47^. This model encompasses the current continuity equations for both electrons and holes, alongside Poisson’s equation:

1 $$\begin{aligned} \frac{\delta (n-N_\text{D}^{+})}{\delta t} = G-R(n,p)-\frac{\nabla \cdot {\textbf {J}}_\text{n}}{q}, \ \ \ \ \ \ \frac{\delta (p-N_\text{A}^{-})}{\delta t} = G-R(n,p)+\frac{\nabla \cdot {\textbf {J}}_\text{p}}{q}, \ \ \ \ \ \ \nabla \cdot {\textbf {E}} = \frac{q}{\varepsilon } (n-p + N_\text{A}^{-}- N_\text{D}^{+}), \end{aligned}$$

where, *q* is the charge of electron, *G* is optical generation rate of the ${\hbox {MoS}}_{2}$ layer, *R* is the recombination rate, $\varepsilon$ is the permittivity of ${\hbox {MoS}}_{2}$, and $N_\text{D}^{+}$ and $N_\text{A}^{-}$ are the ionized donor and acceptor impurity concentrations. $\textbf{J}_\text{n}$ and $\textbf{J}_\text{p}$ are current densities for electrons and holes, which are determined with the drift-diffusion equations, ${\textbf {J}}_\text{n} = qn {\textbf {v}}_\text{n} ({\textbf {E}})+qD_\text{n} \nabla n$ and ${\textbf {J}}_\text{p} = qp {\textbf {v}}_\text{p} ({\textbf {E}})-qD_\text{p} \nabla p$, where, $D_\text{n}={k_BT}\mu _n/q$ and $D_\text{p}={k_BT}\mu _p/q$ are the electron and hole’s diffusion coefficients respectively. ${\textbf {v}}_\text{n} ({\textbf {E}})$ and ${\textbf {v}}_\text{p} ({\textbf {E}})$ are electric-field dependent electron and hole drift velocities respectively. The carrier drift-velocities can be determined using the Caughey–Thomas model^20^, $v_\text{d} = {\mu _\text{0} |{\textbf {E}}|} /({1+\mu _\text{0}|{\textbf {E}}|/v_\text{d,sat}})$, where, *d* is *n* for electrons and *p* for holes, $\mu _\text{0}$ is the low-field carrier mobility and $|{\textbf {E}}|$ is the magnitude of electric field.

The generation rate (*G*) of the ${\hbox {MoS}}_{2}$ layer is determined from the relation $G = {P_0 \alpha }/{A E_{\text{ph}}}$, where $P_0$ is the factor corresponding to incident laser power, $\alpha$ is the absorption coefficient of ${\hbox {MoS}}_{2}$ that is derived from the complex electrical permittivity^6^, *A* is the illuminated surface area of the 2D material layer, and $E_\text{ph}$ is the energy of incident photons. Actual incident power in watts can be calculated by using $P=E\times N_\text{P}/A$, where $N_\text{P}$ is the total number of incident photons during the excitation.

There are three kinds of carrier recombination that we take into account^48^: the recombination due to Shockley–Read–Hall effect, Auger recombination, and radiative recombination. The total recombination is calculated using the following expression:

2 $$\begin{aligned} R(n, p) = (n p - n_i^2) \times \left[ \frac{1}{\tau _\text{p}\times (n+n_i)+\tau _\text{n}\times (p+n_i)} + C_n \times n + C_p \times p + B_r \right] , \end{aligned}$$

where, $n_\text{i}$ is the intrinsic carrier concentration, $\tau _\text{p}$ and $\tau _\text{n}$ are hole and electron lifetimes respectively, $C_n$ and $C_p$ are the Auger recombination rates of electrons and holes respectively, and $B_r$ is the radiative recombination rate.

We have two solvers: a steady-state (static) solver and a transient (dynamic) solver. For the static solver, the potential experienced by the ${\hbox {MoS}}_{2}$ layer is defined by $\psi (x=L) - \psi (x=0)= V_\text{d}-IR_\text{Load}+V_\text{bi}$, where, $\psi (x=L)$ and $\psi (x=0)$ are the potential values at the right and left boundaries of the 1D mesh, $V_\text{d}$ stands for the bias voltage administered at the drain contact, $R_\text{Load}$ denotes the load resistance, $V_\text{bi}$ signifies the inherent potential of the phototransistor, and *I* corresponds to the output current. Initially, neither the value of current *I* nor the final electron and hole concentrations are known. Hence we first apply reasonable guesses and then iteratively apply Newton’s method, determining the consistent values for the currents and charge concentrations. We developed several approaches for making the initial guess since we found that no approach converges in all cases. These include assuming a linear or exponential dependence of the voltage along the phototransistor and assuming that the electron and/or hole concentration peaks at the center of the device or closer to the source or the drain. When none of these approaches work, as happens occasionally, we slightly modify the simulation parameters, including the excitation intensity, bias voltage, and initial doping level, until we obtain a converged solution. We then gradually modify the simulation parameters to return them to the original values.

The dynamic solver starts with the user-defined doping profile and finds the voltages, currents, fields, and carrier concentrations dynamically by solving Eq. (1) using the static solver’s solution as an initial guess. The number of photons absorbed and the number of electron-hole pairs generated and recombined change with time.

**Figure 8:**
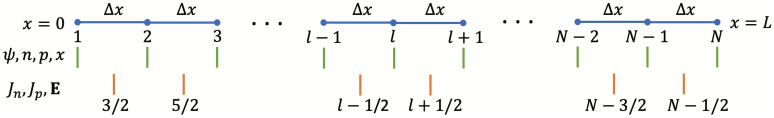
Discretization scheme.

Let us explain this 1D model in detail. We use the implicit Euler method to discretize the drift-diffusion equations in the time domain (*t*) and second-order finite differences to discretize the spatial domain (*x*). Figure 8 schematically shows the mesh that we use to discretize the *x*-dimension. We define the hole density *p*, the electron density *n*, and the electric potential $\psi$, at the integer points in the mesh that are indexed by $l=1,2,...,N$. The current and electric field are defined at intermediate points that are indexed by $l=3/2,5/2,..., N-1/2$.

We approximate the electric field at the half-integer points in the mesh as

3 $$\begin{aligned} E_{l+1/2}= -\left( \frac{\psi _{l+1}-\psi _{l}}{\Delta x}\right) , \end{aligned}$$

where $\psi _l$ is the potential at mesh-point *l*, and we approximate $\partial p/\partial x$ and $\partial n/\partial x$ at the half-integer points as

4 $$\begin{aligned} \left. \frac{\partial p}{\partial x}\right| _{l+1/2}= \left( \frac{p_{l+1}-p_{l}}{\Delta x}\right) \ \ \ \ \ \text{and} \ \ \ \ \ \left. \frac{\partial n}{\partial x}\right| _{l+1/2}= \left( \frac{n_{l+1}-n_{l}}{\Delta x}\right) . \end{aligned}$$

We calculate the currents at the half-integer points by discretizing the drift-diffusion current equations, i.e.,

5 $$\begin{aligned} \begin{aligned} {{\textbf {J}}}_{p,l+1/2}&=qp_{l+1/2} {{\textbf {v}}}_{p,l+1/2}( {{\textbf {E}}})-qD_{p,l+1/2}\left( \frac{p_{l+1}-p_{l}}{\Delta x}\right) ,\\ {{\textbf {J}}}_{n,l+1/2}&=qn_{l+1/2} {{\textbf {v}}}_{n,l+1/2}( {{\textbf {E}}})+qD_{n,l+1/2}\left( \frac{n_{l+1}-n_{l}}{\Delta x}\right) , \end{aligned} \end{aligned}$$

where $p_{l+1/2}=(p_{l+1}+p_{l})/{2}$, $n_{l+1/2}=(n_{l+1}+n_{l})/{2}$, $D_{n,l+1/2}$ and $D_{p,l+1/2}$ are the electron and hole diffusion coefficients at the point ${l+1/2}$, and ${{\textbf {v}}}_{n,l+1/2}$ and ${{\textbf {v}}}_{p,l+1/2}$ are the electron and hole drift velocities at the point ${l+1/2}$.

Using this mesh, we discretize Eq. (1) so that it becomes

6 $$\begin{aligned} \begin{aligned} \frac{n_l^{i+1}-n_l^i}{\Delta t }&=\frac{1}{q}\frac{J_{n,l+1/2}^{i+1}-J_{n,l-1/2}^{i+1}}{\Delta x} {}+G^{i+1}_l-R^{i+1}_l,\\ \frac{p_l^{i+1}-p_l^i}{\Delta t }&=-\frac{1}{q}\frac{{(J_p)}_{l+1/2}^{i+1}-{(J_p)}_{l-1/2}^{i+1}}{\Delta x}{}+G^{i+1}_l-R^{i+1}_l,\\ \frac{1}{{\Delta x}}\left[ {\frac{\varphi _{l+1}^{i+1}-\varphi _{l}^{i+1}}{\Delta x}-\frac{\varphi _{l}^{i+1}-\varphi _{l-1}^{i+1}}{\Delta x}}\right]&=-\frac{q}{\epsilon }\left( {N_{D,l}^+}-{N_{A,l}^-}+p_l^{i+1}-n_l^{i+1}\right) , \end{aligned} \end{aligned}$$

where $n^{i+1}_l$ and $p^{i+1}_l$ are the electron and hole densities at the point *l* and time-step $i+1$, respectively, $G^{i+1}_l$ is the generation rate at the point *l* and time-step $i+1$, $R^{i+1}_l$ is the recombination rate at the point *l* and time-step $i+1$, $\psi ^{i+1}_l$ is the electrostatic potential at the point *l* and time-step $i+1$, and finally ${N_{D,l}^+}$ and ${N_{A,l}^-}$ are the ionized donor and acceptor doping densities at the point *l*. Even though it is obvious, we would like to mention that all the time derivatives in Eq. (6) are set equal to zero for the static solver.

To choose an appropriate time step for stability in convergence, We have followed the principles of Courant condition^49^. Based on this criteria, the time step should follow the relation $\Delta t \le \Delta x/u$, where $\Delta x$ is the mesh size, and *u* is the saturation velocity of electrons/holes.

To calculate the broadband radio frequency (RF) output of the device, we modulate the excitation using the expression $G_\text{in} = G[1+m \sin {(2\pi f_{\text {mod}}t)}]$, where *m* represents the modulation depth and $f_{mod}$ is the modulation frequency. We have selected the total simulation time (*T*) based on the minimum modulation frequency in RF output power calculation, i.e., $T = 10/\text{min}(f_{\text {mod}})$ allows us to investigate how fields, currents, and charges change over 10 periods. Phase noise of the photodetector is a crucial metric for many applications where precise timing and synchronization are critical. In this work, we determine the phase noise of the outlined phototransistor using the identical methodology proposed by Mahabadi et al.^43^.

### 2D material based phototransistor modeling

It is known that the defects in 2D materials (vacancies, substitutions, and grain boundaries) can create localized electronic states within the band gap of the 2D material, leading to the formation of donor or acceptor states. Donor states provide additional electrons, leading to *n*-type doping, while acceptor states create holes, resulting in *p*-type doping. For the monolayers of ${\hbox {MoS}}_{2}$, the most common defect is sulfer vacancies, which lead to *n*-type doping^50^. Hence in this work, we assume that the ${\hbox {MoS}}_{2}$ monolayer is intrinsically *n*-doped and its initial doping density is equal to the density of the intrinsic defects (or traps), $N_\text{traps}$, where $N_\text{traps}$ is set equal to $10^{10}$ cm$^{-2}$. The applied gate voltage ($V_\text{g})$ can change this initial doping density if it is larger than a threshold value, *i.e.*, if $V_\text{g}>V_\text{th}$, then we calculate the doping density with the expression of $n_\text{s} = {\varepsilon _\text{ox} (V_\text{g}-V_\text{th})}/{t_\text{ox}}$, where $V_\text{th} = N_\text{traps} t_\text{ox} / \varepsilon _\text{ox}$, $t_\text{ox}$ is the thickness of the oxide layer, and $\varepsilon _\text{ox}$ is the permittivity of the oxide layer.

The resistance, $R_\text{Load}$, given by $R_\text{Load} = \sqrt{\rho _i \times R_\text{sh}}$, where $R_\text{sh}$ is the sheet resistance of the ${\hbox {MoS}}_{2}$ monolayer, $R_\text{sh}=1/{qn\mu _n(T)A}$, and $\rho _i$ is the interfacial resistance between the contact and the ${\hbox {MoS}}_{2}$, with *T* being the temperature and $\mu _n$ is the electron low-field mobility, as discussed in Supplementary Materials. We calculate the interfacial resistance using an empirical formula ($\rho _i = 1/[(T-100)/107]^3$) derived from the experimental data^51^.

## Material modeling

A key element of our material modeling was accurately solving for the drift and diffusion in the ${\hbox {MoS}}_{2}$ monolayer. This monolayer is responsible for carrying the photo-generated carriers, leading to the emergence of photocurrent. As previously mentioned, the direct energy bandgap of the monolayer ${\hbox {MoS}}_{2}$ is $\sim 1.8$ eV. This bandgap can be calculated more accurately based on simulation parameters by using the Varshni equation^52^, $E_\text {g}(T)=E_\text {g}(0)-\alpha T^2/((T+\beta ))$, where $E_\text {g}(0)=1.95$ eV, $\alpha =5.9\times 10^{-4}$ eV/K, and $\beta =430$ K in our simulations. The bandgap value at room temperature is $\sim$ 1.87 eV. In the literature, a wide range of values have been used for the effective masses of electrons ($m_e$) and holes ($m_h$). We use $m_e=0.35m_0$ and $m_h=0.5m_0$ in this work^53^. Another crucial attribute of ${\hbox {MoS}}_{2}$ is its electron affinity ($\chi _i$), which is the energy released when an electron associates with a neutral atom to create a negatively charged ion. Gong et al. conducted numerical computations, yielding $\chi _i = 4.27$ eV for the monolayer ${\hbox {MoS}}_{2}$^54^, a value that we have adopted for our simulations. For the numerical assessment of carrier recombination, we require coefficients for both radiative recombination and non-radiative Auger recombination. We adopted the following values: $10^{-7} \; \mathrm {cm^{3}/s}$ and $10^{-24} \; \mathrm {cm^{6}/s}$, respectively^55^. To compute the density of holes and electrons before and after the illumination, the density of states (DOS) in the conduction and valence bands are required. In this work, the effective density of states is calculated using the expression $N_\text{C} = 2\times (2\pi m_e^*kT/h^2)^{3/2}$ for the conduction band and $N_\text{V} = 2\times (2\pi m_h^*kT/h^2)^{3/2}$ for the valence band^56^. The calculated effective density of states at room temperature equals $3.76 \times 10^{11}$ cm$^{-2}$ for the conduction band and $5.76 \times 10^{11}$ cm$^{-2}$ for the valence band. To calculate recombination rates, we require electron and hole lifetimes, which are assumed equal to 1 ns and 10 ns respectively. Finally, to solve the drift-diffusion equations, we also must set the saturation velocity of carriers. In this work, we set the electron’s saturation velocity ($v_\mathrm{{n,sat}}$) as $4.2\times 10^6 \; \mathrm {cm/s}$ and the hole’s saturation velocity ($v_\mathrm{{p,sat}}$) as $1\times 10^7 \; \mathrm {cm/s}$^57^. We summarize the material parameters for monolayer ${\hbox {MoS}}_{2}$ in Table 2. The complex electrical permittivity of ${\hbox {MoS}}_{2}$ is calculated using the numerical model proposed by Mukherjee et al.^6^. This model first determines bright excitonic states of the monolayer ${\hbox {MoS}}_{2}$ using an atomistic method that takes local temperature and Fermi level into account. Then we calculate the complex electric permittivity via a hybrid Lorentz–Drude–Gaussian model as a function of wavelength. For the refractive indices of SiO$_2$ and Si, we use numerical models developed by others^58,59^.

**Table 2:** Material parameters of ${\hbox {MoS}}_{2}$ at $T=300$ K used in our simulations.

Parameter name	Symbol	Value
Energy bandgap^52^	$E_\text{g}$	1.87 eV
Electron’s effective mass^53^	$m_\text{e}^*$	0.35$m_0$
Hole’s effective mass^53^	$m_\text{h}^*$	0.50$m_0$
Electron affinity^54^	$\chi _i$	4.27 eV
Radiative recombination coefficient^55^	$B_\text{r}$	$10^{-7} \mathrm \; cm^3/s$
Auger coefficient^55^	$C_\text{n},C_\text{p}$	$10^{-24} \mathrm \; cm^6/s$
Density of states in conduction band^56^	$N_\text{C}$	$3.76 \times 10^{11} \mathrm \; cm^{-2}$
Density of states in valence band^56^	$N_\text{V}$	$5.76 \times 10^{11} \mathrm \; cm^{-2}$
Hole saturation velocity^57^	$v_\text{p,sat}$	$1 \times 10^7 \mathrm \; cm/s$
Electron saturation velocity^57^	$v_\text{n,sat}$	$4.2 \times 10^6 \mathrm \; cm/s$
Electron lifetime^38^	$\tau _\text{n}$	$1 \times 10^{-9} \mathrm \; s$
Hole lifetime^38^	$\tau _\text{p}$	$1 \times 10^{-8} \mathrm \; s$

$m_0$ is the electron mass.

All the codes are implemented using MATLAB.

### Supplementary Information

Supplementary Information.

## Data Availability

The datasets generated during and analyzed during the current study are available from the corresponding author on reasonable request.
