# Supplementary material for: Study of an MoS2 phototransistor using a compact numerical method enabling detailed analysis of 2D material phototransistors
Source: Sci Rep. 2024 Jul 3;14:15269. doi: 10.1038/s41598-024-66171-1 (PMC11222441; doi:10.1038/s41598-024-66171-1)
Supplement: Supplementary file 1 — Supplementary Information. [file 41598_2024_66171_MOESM1_ESM.pdf]

Supplementary Materials

Inverse Fermi-Dirac Integral

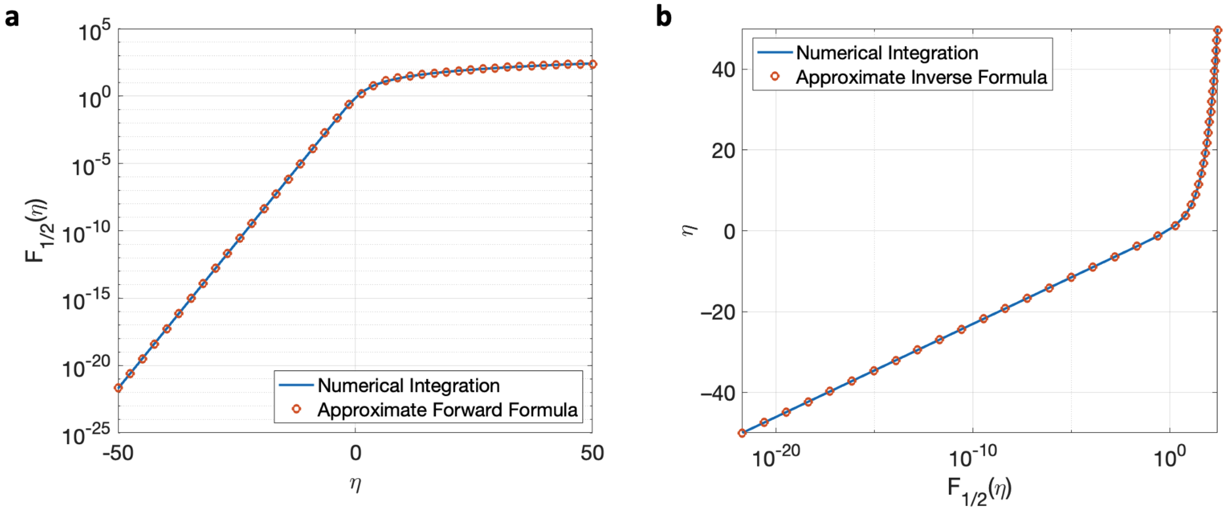

Supp. Fig. 1. (a) $F_{1/2}(\eta)$ values calculated with numerical integration of Eq. (1) and using the approximate formula (2) for $-50 < \eta < 50$, (b) Inverse Fermi calculation, where the approximate results are obtained with Eq. (3).

The Fermi-Dirac integral,

$$F_{1/2}(\eta) = \frac{1}{\Gamma(3/2)} \int_0^\infty \frac{t^{1/2}}{e^{t-\eta}+1}\, dt, \tag{1}$$

is a mathematical function that describes the distribution of electrons in a system at thermodynamic equilibrium, based on Fermi-Dirac statistics. This well-known integral plays a crucial role in semiconductor physics.

Calculating $F_{1/2}(\eta)$ for a given η, where η is the ratio of the Fermi energy to the product of the Boltzmann constant and temperature, via numerical integration is not a trivial task but the inverse operation (finding η for a given $F_{1/2}$ value) is. Hence we have created two simple yet accurate formulas, Eqs. (2) and (3), to compute these forward and inverse operations efficiently.

$$F_{1/2}(\eta) \approx 10^{g(\eta)}, \quad \text{where} \quad g(\eta) = \frac{-10.39\eta^4 + 1630\eta^3 - 27360\eta^2 + 5.723 \times 10^5\eta - 3.352 \times 10^5}{\eta^4 + 92.29\eta^3 + 8472\eta^2 + 16330\eta + 1.8 \times 10^6}. \tag{2}$$

$$\eta \approx \frac{2.309x^5 - 23.17x^4 + 99.36x^3 - 181.2x^2 + 213.9x + 27.2}{x^4 - 10.17x^3 + 41.76x^2 - 85.66x + 78.43}, \quad \text{where} \quad x = \log_{10}(F_{1/2}(\eta)). \tag{3}$$

As shown in Supp. Figs. 1 (a) and (b), these formulas provide us with very accurate approximations of both sides of Eq. (1) enabling us to calculate the energy band diagram of the device.

Additional Numerical Analysis

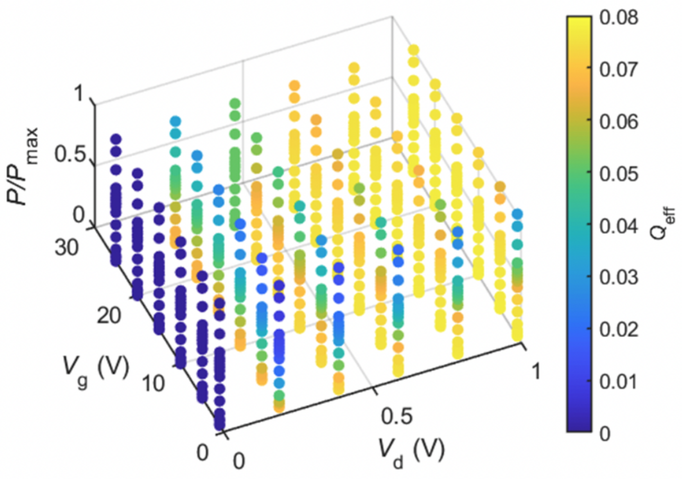

Supp. Fig. 2. Quantum efficiency (Q_{eff}) as functions of gate voltage (V_g), drain-to-sourve voltage (V_d), and normalized incident power P/P_{max}. $\lambda = 561$ nm. Observation: Q_{eff} increases with increasing voltages and decreasing incident power.

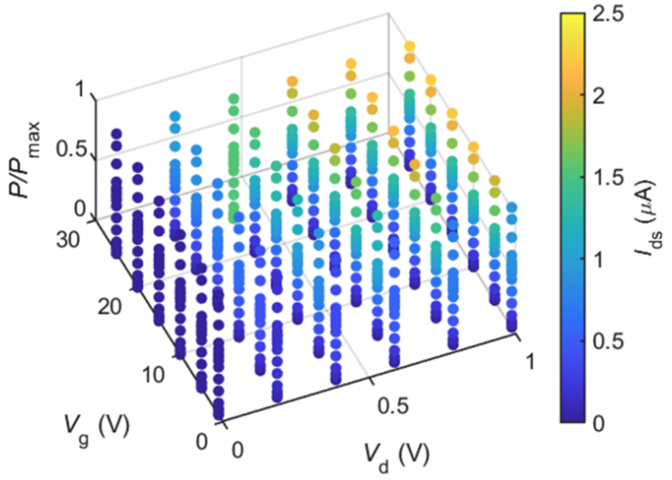

Supp. Fig. 3. Output current as functions of gate voltage (V_g), drain-to-sourve voltage (V_d), and normalized incident power P/P_{max}. $\lambda = 561$ nm. Observation: Output current increases with increasing voltages and incident power.

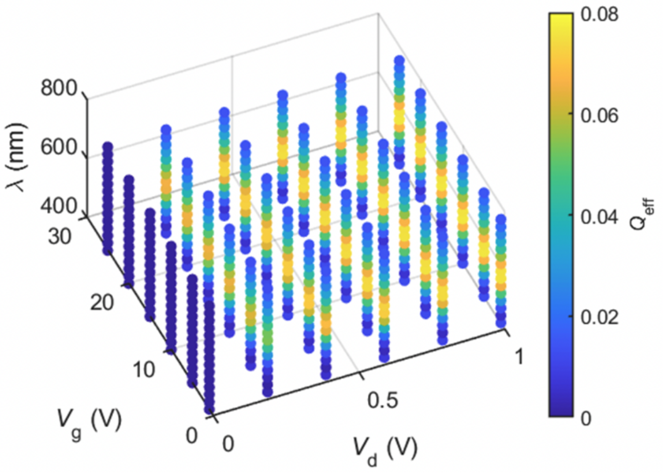

Supp. Fig. 4. Quantum efficiency as functions of gate voltage (V_g), drain-to-sourve voltage (V_d), and wavelength (λ). $P_{\rm inc} = 2$ nW. Observation: $Q_{\rm eff}$ is highest near 560 nm and it increases with increasing voltages.

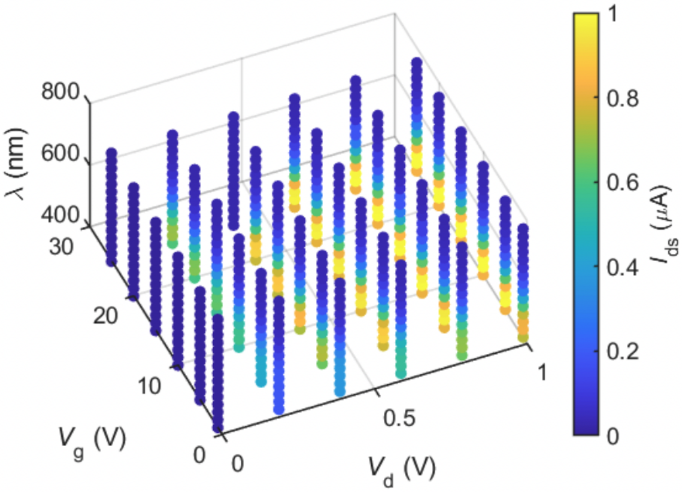

Supp. Fig. 5. Output current as functions of gate voltage (V_g), drain-to-sourve voltage (V_d), and wavelength (λ). $P_{\rm inc} = 2$ nW. Observation: Output current is highest near 425 nm and it increases with increasing voltages.

Plane Wave Propagation in Multi-Layered Media

To determine the electric field intensity inside the monolayer MoS_2, we use the plane wave propagation in multi-layered media formalism. In this approach, the field intensities in each layer are calculated recursively, starting from the first layer, where the intensity of the forward traveling wave is known from the incident power, P. Figure 6 (b) shows the electric field intensity in the middle of the MoS_2 layer as a function of the excitation wavelength. We observe that the intensity reaches its maximum value around 561 nm and makes two dips around 420 nm and 830 nm.

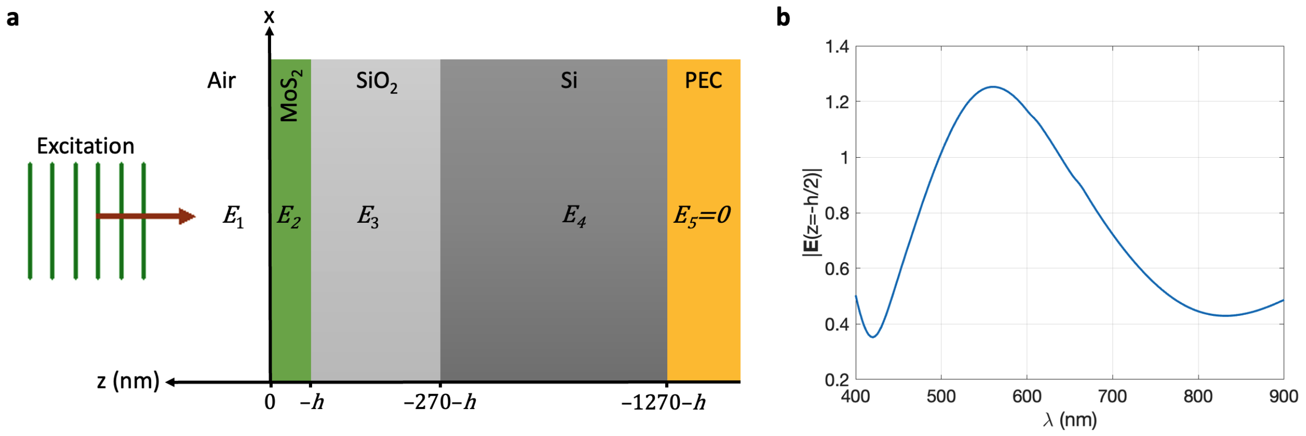

Supp. Fig. 6. (a) The phototransistor consists of 4 layers: 0.65 nm thick MoS_2, 270 nm thick SiO_2, 1 μ thick Si, and perfectly electrical conductor (PEC). (b) The electric field intensity in the middle of the MoS_2 layer as a function of the excitation wavelength.

General Formulation

Assume a multi-layered medium with $N+1$ planar layers aligned parallel to the xy-plane. Each layer is defined with its electrical permittivity ($\epsilon_\ell = \epsilon_0\epsilon_{r,\ell}$), magnetic permeability ($\mu_\ell = \mu_0\mu_{r,\ell}$), thickness ($h_\ell$) for $\ell = 0, 1, \cdots, N$, and $h_0 = h_{N+1} = \infty$, and ϵ_0 and μ_0 are the electrical permittivity and magnetic permeability of vacuum. When this multi-layered medium is excited from the first layer with a transverse-electric (TE) or transverse-magnetic (TM) wave, the fields in each layer can be written as a sum of two components: forward traveling and backward traveling. We can define a **global** reflection coefficient as the ratio of these two parts at the interfaces. For example, the field in layer i can be written as

$$\phi_i = A_i[e^{-jk_{iz}(z-z_i)} + \widetilde{R}_{i,i+1}e^{jk_{iz}(z-z_i)}] \tag{4}$$

where $\widetilde{R}_{i,i+1}$ is called the global reflection coefficient between layer i and layer $i+1$, and

$$\phi_i = \begin{cases} E_{iy}, & \text{for TE}^z \\ H_{iy}, & \text{for TM}^z \end{cases} \qquad A_i = \begin{cases} E_{i0}e^{-jk_x x}e^{-jk_{iz}z_i}, & \text{for TE}^z \\ H_{i0}e^{-jk_x x}e^{-jk_{iz}z_i}, & \text{for TM}^z \end{cases} \tag{5}$$

where $z_N = z_{N-1}$ is assumed but this assumption does not affect the results (in reality, $z_N \to \infty$). The two terms in (4) will be denoted as the FW_1 and BW_1 waves, corresponding to forward-traveling and backward-traveling waves, respectively, in the ith layer.

Similarly, the field in layer $i+1$ can be written as in (4)

$$\phi_{i+1} = A_{i+1}[e^{-jk_{i+1,z}(z-z_{i+1})} + \widetilde{R}_{i+1,i+2}e^{jk_{i+1,z}(z-z_{i+1})}] \tag{6}$$

The two terms in (6) will be denoted as the FW_2 and BW_2 waves in the $(i+1)^{st}$ layer.

Now consider the field in layer $i+1$ at the interface $z = z_i$. The forward-traveling wave FW_2 has two contributions: (i) the local transmission ($T_{i,i+1}$) of FW_1, and (ii) the local reflection ($R_{i+1,i}$) of BW_2. Therefore from (4) and (6) we have

$$A_{i+1}P_{i+1}^{-1} = T_{i,i+1} \cdot A_i + R_{i+1,i} \cdot A_{i+1}\widetilde{R}_{i+1,i+2}P_{i+1} \tag{7}$$

where $P_{i+1} = \exp[-jk_{i+1,z}(z_{i+1} - z_i)]$ is the "propagator" in layer $i + 1$.

Similarly, consider the backward-traveling wave in layer i at the interface $z = z_i$. The backward-traveling wave BW_1 also has two contributions: (i) the local transmission ($T_{i+1,i}$) of BW_2, and (ii) the local reflection ($R_{i,i+1}$) of FW_1. Therefore,

$$A_i \widetilde{R}_{i,i+1} = T_{i+1,i} \cdot A_{i+1} \widetilde{R}_{i+1,i+2} P_{i+1} + R_{i,i+1} \cdot A_i \tag{8}$$

Solving (7) and (8) yields

$$\widetilde{R}_{i,i+1} = R_{i,i+1} + \widetilde{R}_{i+1,i+2} T_{i+1,i} P_{i+1}^2 \widetilde{T}_{i,i+1} \tag{9}$$

$$A_{i+1} = A_i \widetilde{T}_{i,i+1} P_{i+1} \tag{10}$$

where we have assumed that $z_N = z_{N-1}$ (thus $P_N = \exp[-jk_{Nz}(z_N - z_{N-1})] = 1$), and

$$P_i = e^{-jk_{iz}(z_i - z_{i-1})} \tag{11}$$

The global transmission coefficient is

$$\widetilde{T}_{i,i+1} = \frac{T_{i,i+1}}{1 - R_{i+1,i} \widetilde{R}_{i+1,i+2} P_{i+1}^2} \tag{12}$$

In the above, P_i is called the propagator for layer i since it denotes the propagation from z_{i-1} to z_i. Equations (9) and (10) give the recursive relations for $\widetilde{R}_{i,i+1}$ and A_{i+1}. Obviously, the "initial" conditions are

$$\widetilde{R}_{N-1,N} = R_{N-1,N} \tag{13}$$

$$A_1 = \begin{cases} E_{10} e^{-jk_x x} e^{-jk_{1z} z_1}, & \text{for TE}^{\text{z}} \\ H_{10} e^{-jk_x x} e^{-jk_{1z} z_1}, & \text{for TM}^{\text{z}} \end{cases} \tag{14}$$

From (13) and (9), we can find $\widetilde{R}_{N-2,N-1}, \cdots, \widetilde{R}_{1,2}$. Similarly, from (14) and (10), we can find $A_2, \cdots, A_N$.

To find the transmission wave amplitude, we use (14) and (10) repeatedly and obtain

$$A_N = A_1 \prod_{\ell=1}^{N-1} [\widetilde{T}_{\ell,\ell+1} P_{\ell+1}] = \widetilde{T}_{1N} A_1 \tag{15}$$

where $P_N = 1$, and

$$\widetilde{T}_{1N} = \prod_{\ell=1}^{N-1} [\widetilde{T}_{\ell,\ell+1} P_{\ell+1}] \tag{16}$$

is called the global transmission coefficient between layer 1 and layer N.

Given the incident field, then the field in all layers can be obtained by (16). Note that if the last layer is air, then $\widetilde{R}_{N,N+1} = 0$ and if it is a PEC, then $\widetilde{R}_{N,N+1} = -1$.
